# The Postural Control Indexes during Unipodal Support in Patients with Idiopathic Scoliosis

**DOI:** 10.1155/2020/7936095

**Published:** 2020-07-04

**Authors:** Aneta Dąbrowska, Marzena A. Olszewska-Karaban, Anna K. Permoda-Białozorczyk, Dominika A. Szalewska

**Affiliations:** ^1^Department of Rehabilitation Medicine, Medical University of Gdansk, Al. Zwycięstwa 30, 80-219 Gdansk, Poland; ^2^Gdansk College of Health, Pelplińska 7, 80-335 Gdansk, Poland; ^3^Biomed Rehabilitation Center, Dębinki 7d, 80-294 Gdansk, Poland; ^4^Department of Health and Natural Science, Gdansk University of Physical Education and Sport, Kazimierza Górskiego 1, 80-336 Gdańsk, Poland

## Abstract

Proper posture provides the best balance and body stability at minimal muscular effort. It is constantly controlled by the central nervous system, which integrates the stimuli from the proprioceptors (deep feeling sensors), vision receptors, and balance receptors through the subcortical structures. The main purpose of the study was to describe single stance stability and its correlation with the degree of scoliosis and trunk rotation among patients suffering from idiopathic scoliosis and in the control group without scoliosis. The study included 80 patients (69 girls and 11 boys) and 40 healthy children without scoliosis (21 girls and 19 boys). The Cobb angle technique was used to determine the magnitude of the deformity. All subjects were divided into three subgroups according to Bogdanov's classification. Single stance stability with eyes open and eyes closed was assessed with an electronic postural station—Delos Postural Proprioceptive System (DPPS). In case of multiple group comparisons for variables with normal distribution ANOVA with Scheffe, post hoc test was used or Kruskal-Wallis test was used as the nonparametric equivalent. The relationship between the two continuous variables was investigated using either Pearson product-moment correlation or Spearman's rank correlation. In all these calculations, the statistical significance level was set to *p* < 0.05. The single stance test showed a significant difference between the stability index with eyes open and stability index with eyes closed in study and control groups. The character of these alterations is influenced by the degree of trunk rotation. The degree of scoliosis according to Bogdanov classification does not determine the decrease in stability indexes. In summary, significantly lower values of the stability index during one-leg standing with eyes closed indicated balance impairment, which is mainly connected with inadequate functioning of the proprioceptive system.

## 1. Introduction

Proper posture provides the best balance and body stability at the minimal muscular effort. It is constantly controlled by the central nervous system (CNS), which integrates the stimuli from the proprioceptors (deep feeling sensors), vision receptors, and balance receptors through the subcortical structures [1]. Proprioception refers to the signals coming from muscles, tendons, and joints of the human being, which inform about the arrangement of particular parts of body from one to another. It enables creation of a body diagram based on the feeling of mutual arrangement of its elements and the movement of its segments. In patients with scoliosis, this pattern may be disturbed or abnormal. Though proprioception, sense of balance, and vision inputs continually complement each other, it is proprioception that plays the major role in the postural control system [2–4].

Still, even the slightest disturbance in the action of any of the above systems may affect the whole process of postural control and human balance. The process which regulates postural and body balance reflexes may also be affected by scoliosis.

Idiopathic scoliosis is a three-dimensional deformation of the spine. In the sagittal plane, it involves either increased or decreased physiological curvature of the spine; in the frontal plane, vertebrae are inclined to the side; and in the transverse plane, vertebrae are rotated. In spite of developing diagnostic tools and advanced medical and surgical treatment of scolioses, the ethology of the condition still remains unknown. Research indicates a significant contribution of the CNS in the etiology of scolioses [5–7]. Idiopathic scoliosis in adolescents is a common disease with an incidence of 0.47-5.2% in current literature [8]. Despite this, there is still too little research in the literature that would examine the functioning of the posture control system in relation to changes in spinal curvature disorders.

The aim of the study was to determine the postural control index, the proprioceptive control index, and the role of visual information during unipodal support in patients with idiopathic scoliosis. The hypothesis in this study states that the degree of scoliosis and trunk rotation correlates with the indexes of postural stability and proprioception in patients with idiopathic scoliosis. The occurrence of three-dimensional deformation of the spine has been significantly related to the deterioration of the body balance parameters.

## 2. Materials and Methods

### 2.1. Subjects

The postural control system was assessed on 120 subjects: the study group of 80 patients with scoliosis (69 girls and 11 boys) aged 11-18 (average age: 13.96; SD 1.80) and the control group of 40 healthy children (21 girls and 19 boys) aged 11-18 (average age: 14.11; SD 2.08). The characteristics of the subjects are summarized in Table 1. This study was conducted in a private rehabilitation center in Gdańsk (Poland) between January 2016 and January 2017. Prior to tests, a medical history questionnaire was administered to obtain data on the subjects' health status. The subjects of the study were volunteers that met the inclusion and exclusion criteria. A consecutive sampling technique was applied for the recruitment.

Inclusion criteria to the study group were idiopathic scoliosis with a Cobb angle between 10 and 55 and no previous spine surgery or other musculoskeletal disorder.

Exclusion criteria both for the study and control group were the subjects with nervous system diseases, sensory integration disorders, condition after lower limb fractures, and injuries in the year preceding the study or with lower limb abbreviations. Moreover, subjects with any posture defects and scoliosis were also excluded from the control group.

The number of study participants was determined on the basis of a pilot study on 24 people with idiopathic scoliosis, whose body posture control results have been compared to the standards specified by the device producer. The 40 age-matched control children were recruited from surrounding schools in Gdańsk (primary schools, junior and senior high schools). The study group was divided into three subgroups according to Bogdanov's classification (Table 2) [9] and in relation to the angle of trunk rotation (Table 3). 29 participants from the study group had not received conservative treatment before. 51 were undergoing conservative treatment: 42 participants with a brace and kinesiotherapy and 9 only kinesiotherapy. The authors of this study obtained the consent of the Independent Bioethics Committee for Scientific Studies at the Medical University of Gdansk as of July 02, 2015, resolution number NKBBN/306/2015 to conduct the study in the scope of this publication. Additionally, every legal guardian of the minor patient expressed their written informed consent to minor's participation in the study performed.

A mean value of Cobb angle was 28.1 ± 11.63 degrees, with a Cobb angle of 10 degrees regarded as a minimum angulation to define scoliosis and a maximum value of 55 degrees. In 23 participants with scoliosis, the primary curvature was located in the thoracolumbar spine, in 38 in the thoracic spine, and in 19 in the lumbar spine. In order to determine the influence of the scoliosis value measured according to the Cobb angle technique on the decreased level of postural and proprioceptive control, the subjects were divided into three subgroups according to Bogdanov's classification (Table 2).

The subgroups were homogenous with respect to the subjects' age, *p* > 0.05.

A mean value of trunk rotation at the level of the apical vertebra of the primary curve was 8.24 ± 3.48 degrees, with a minimum value of 2 degrees and maximum of 20 degrees. Three subgroups of subjects depending on the spine deviation advancement stage were selected subsequently. The subgroups were homogenous with respect to the subjects' age, *p* > 0.05. Characteristics of the participants are shown in Table 3.

### 2.2. Methods

#### 2.2.1. Procedures

**Figure 1:**
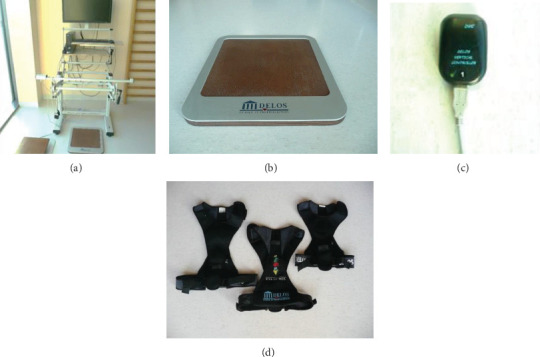
Delos Postural Proprioceptive System: (a) the postural proprioceptive station; (b) wooden research station (Delos Equilibrium Board); (c) DVC vertical controller (Delos Vertical Controller); (d) vests used for DVC fastening (material from the “Wyspa” Therapy Center in Gdańsk, Poland).

The study group was diagnosed in a physical examination followed by an X-ray examination in the posterior-anterior view (P-A) obtained in standing position. The Cobb angle technique was used to determine the magnitude of the deformity, involving measuring the angle between the lines parallel to the border vertebral bodies of the structural curve [10]. All patients showed the features of three-dimensional deformation in the axis of the spine, confirmed by angle of trunk rotation examination (ATR) using a scoliometer during the Adams Forward Bend Test [11, 12]. The starting position for the study was an upright position with arms outstretched forward and hands joined flat. The patients performed a slow forward bend so that the torso was parallel to the ground during the rotation reading. The measurement was taken at the place of the greatest deformation across the long axis of the spine. The assessment of the postural control index and proprioceptive control index was performed by means of Delos Postural Proprioceptive System (DPPS 6.0, Gdańsk, Poland), which is an electronic postural proprioceptive station connected to a personal computer equipped with a special software [13, 14] (see Figure 1).

#### 2.2.2. Instruments

Delos Postural Proprioceptive System makes it possible to evaluate the posture control system while standing on one leg with eyes alternately open and closed and allows for controlled training of posture stability and the balance of lower limbs, spine, and pelvis, using visual feedback elements. The single stance test (SST) was conducted with the subjects standing on the left leg first and then on the right, with the eyes open and closed. SST comprised six trials, 20 s each (two trials with open eyes (OE) and four with closed (CE)). Elements of the postural stability control system Delos enable to assess the deep feeling in the extremity tested by means of the static wooden station DEB (Delos Equilibrium Board), electronic postural reader DVC (Delos Vertical Controller) applied on the subject's sternum, and a metal bar with infrared sensor DPA (Delos Postural Assistant) for support in case of lack of postural control (see Figure 1). DPA informed how often the subject had to support on the horizontal bar during the whole test in order to minimize the fall risk or regain vertical control balance. As a precaution of falling, the subjects could touch the bar placed in front of them to regain vertical control rapidly. DPA consists of an infrared sensor attached to the adjustable bar placed in front of a subject. The sensor allows to indicate the longest period of the balance kept by the subject with the use of the bar. DVC is a two-dimensional accelerometer unit measuring mean trunk inclination amplitude in the frontal and sagittal plane with respect to the midline (PIxy—average postural instability or amplitude of the postural cone) (see Figure 1). DVC deviations range is 0-30 centimeters. The conjunctive element of Delos is a Postural System Analyzer (PSA). PSA is a software which allows to visualize and analyze input data in real time simultaneously with those from DVC, DEB, and DPS.

#### 2.2.3. The Postural Control Index: The Evaluation of the Postural Control Index Was Carried Out by Means of the Stability Index with the Eyes Open (SI OE)

The stability index (SI) was determined by the trunk inclination on the grounds of the assessment of postural instability and is capable of ranking all kinds of performances from the highest to the lowest level. SI is a score from 0 to 100 percentage [15]. The system autonomy is the time when the subject is not leaning with their hands against the bar. Individuals with a very well-functioning postural system exhibit high stability of body position in both static and dynamic loss of balance situations, keeping the head and trunk in an almost stable position. That was evidenced by the low result of the average postural instability and full autonomy of the system expressed in the absence of the support use in the event of loss of body balance.

#### 2.2.4. The Proprioceptive Control Index: The Evaluation of the Proprioceptive Control Index Was Carried Out by Means of the Stability Index with the Closed Eyes (SI CE)

A high value of the proprioceptive control index is characteristic of a proper balance, maintained during single-leg standing without visual control. A decrease in the proprioceptive control system manifests at first by the increase in the amplitude of the postural cone (PIxy) and next by the increase in applying the precautionary strategy, i.e., supporting by leaning against the bar. Afterward, if such need exists, the amount of necessary support is increasing and the amplitude of the postural cone decreases. This situation can occur during trials with both closed and open eyes. The stability index during the trial with closed eyes is the result of the effectiveness of the proprioceptive system, even though the simultaneous activation of the vestibular system cannot be excluded [15].

#### 2.2.5. Statistical Analysis: All Calculations Have Been Carried Out by Means of Microsoft Excel Spreadsheet and STATISTICA, StatSoft, Inc., ver. 12.0 Statistical Package (Data Analysis Software System)

In the statistical description of quantitative data, classical measures of location such as arithmetic means and median and measures of variation such as standard deviation and range were used. The normality of distribution of the variables and variance equality of a studied feature in groups were tested by the use of appropriate Shapiro-Wilk's test and a variance equality test. In order to compare groups in pairs for quantitative data, *t*-test or Mann-Whitney test was used with respect to the type of distribution of the variables tested. In case of multiple group comparisons for variables with normal distribution ANOVA with Scheffe, post hoc test was used or Kruskal- Wallis test was used as the nonparametric equivalent of one-way ANOVA, with Dunn's test as post hoc test. When comparing pairwise repeated measurements, Wilcoxon test was used. The relationship between the two continuous variables was investigated using either Pearson product-moment correlation or Spearman's rank correlation. In all these calculations, the statistical significance level was set to *p* < 0.05.

## 3. Results

The obtained results indicate significant differences between the mean of the postural control index SI OE (stability index in trials with eyes open) and the mean of the proprioceptive control index SI CE (stability index in trials with eyes closed) in the control group (*p* ≤ 0.001) and in the group with idiopathic scoliosis (*p* ≤ 0.001). Closure of eyes determined deterioration of the body balance parameters studied regardless whether scoliosis was present or not.

**Figure 2:**
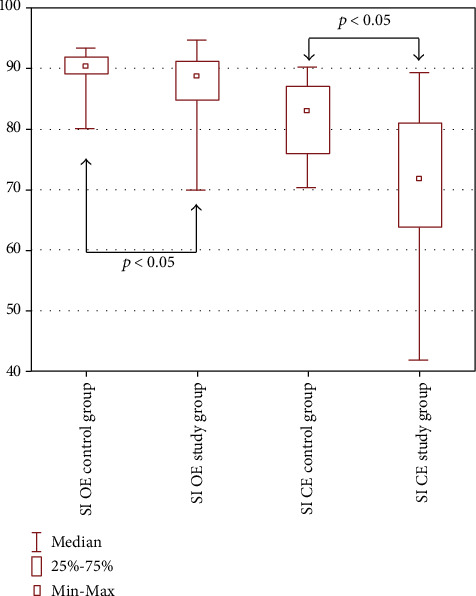
Graphical comparison of the mean stability index between the control and the scoliosis (study) group. SI OE: stability index in trials with open eyes/postural control index; SI CE: stability index in trials with closed eyes/proprioceptive control index.

A slight but significant difference was observed in the result of SI OE between the control and the study groups, *p* < 0.05. For closed eyes trials, the differences in the average level of the stability index (SI CE) were more significant. Patients with scoliosis achieved statistically lower levels of proprioceptive control index compared to the control group, *p* ≤ 0.001 (see Figure 2).

Depending on the scoliosis angle measured according to the Cobb angle technique and Bogdanov classification (*n* = 80), the obtained results showed a significant difference in stability index in trials performed with open and closed eyes in all groups, *p* ≤ 0.001.

**Figure 3:**
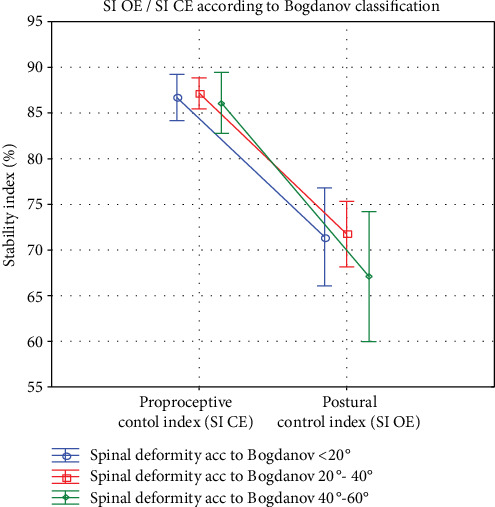
Graphic comparison of the mean values of the stability indexes in the groups with open and closed eyes. SI OE and SI CE with respect to scoliosis value according to Bogdanov. SI OE: the stability index in open eyes trials; SI CE: the stability index in closed eyes trials.

However, irrespective of the fact whether the eyes were open or closed, the scoliosis degree according to the Bogdanov classification did not have influence on the decrease in stability indexes, *p* > 0.05 (see Figure 3). Figure 3 shows that in the closed eyes trials, scoliosis measured according to the Cobb angle technique and amounting to 20-40° and 40-60° were both in the falling tendency; however, the difference between these groups was insignificant.

**Figure 4:**
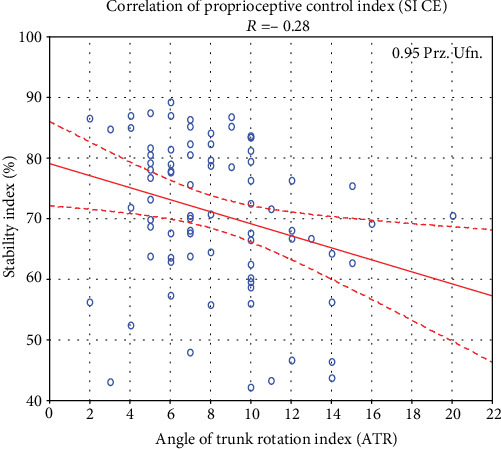
The diagram of correlation between ATR index and the mean stability index in the closed eyes trials (*n* = 79). ATR: angle of trunk rotation; SI CE: stability index in closed eyes trials.

Relationships examined by Spearman rank correlations did not indicate significant correlations between ATR and SI OE (*R* = −0.07, *p* > 0.05). Statistically significant negative correlations were noted between SI CE and the value of the angle of trunk rotation (*R* = −0.29, *p* < 0.05) (see Figure 4). In trials with closed eyes, stability index SI CE (the proprioceptive control index) and its value depend on the spine rotation angle. Furthermore, the difference between SI CE results occurs in values of the angle of trunk rotation ATR > 11° and ATR < 5° (*p* < 0.05) and ATR 6-10° (*p* < 0.05), which is shown in Table 4.

## 4. Discussion

The single stance test was aimed at determining the value of the stability index in patients suffering from idiopathic scoliosis and in the control group. Moreover, the authors attempted to examine the influence of the magnitude of scoliosis determined by the Cobb angle technique and the angle of trunk rotation on the functioning of the postural control system in the subjects. Test results may allow to determine these clinical variables which could affect the postural control system functioning. A hypothesis was made that among patients with diagnosed scoliosis, the postural control index and the proprioceptive control index were significantly lower compared to those without scoliosis. What is more, the degree of scoliosis and grade of trunk rotation correlate with the deterioration of the level of the indexes.

In literature, there has been a growing interest in the study of systems responsible for balance and control of body posture. According to Dayer et al., irregularities in posture control concern primarily the vestibular and the proprioceptive systems and usually occur during their maturation [16]. Moreover, Liu et al. have used tests activating the proprioceptive system and the eye, with the use of magnetic resonance imaging, and proved that there are significant average volume differences in 22 areas of the brain between patients with diagnosed juvenile idiopathic scoliosis and a healthy group [17]. Gauchard et al. observed that the deformity of the spine causes distortion of the body schema [18]. Recently, Fortin et al. noticed that patients with AIS showed different electrocortical brain dynamics in the sensorimotor cortex to maintain the level of balance control as age-matched controls [19]. The main change in electrocortical dynamics is a significant increase in the frequency power and greater alpha, beta, and attenuation gamma frequency power when proprioception is changed. These results suggest that the assessment of bark dynamics may be relevant to investigate the function of sensorimotor integration in AIS. Furthermore, another researcher noticed compensatory changes in the control of the motions of the body segments between the gait cycles of the convex and concave sides [20].

The fact that the single-limb support period accounts for 80% of the gait cycle at normal walking speed while the double-support period accounts for 20% [21] suggests an important role for single stance stability in the safety of walking.

In our study, the postural control system was assessed in single-leg support with eyes alternately open and closed. The stability index of EO trials was taken as an indicator of postural control while the stability index of EC trials was considered an indicator of proprioceptive control and of its effectiveness as the primary stabilizer of posture.

According to Riva, it is sufficient to conduct a static single stance test to evaluate the postural strategy responsible for restoring the disturbed body balance and determine compensation mechanisms [3, 13, 15, 22]. During proper control of body posture, high values of stability index in EC trials correspond to refined proprioceptive control, because they are the expression of effective proprioceptive reflexes enabling to stabilize the subject instantly, before the vestibular responses can be activated. The vestibular organ is much more active when information from the receptors of deep sensation and the organ of vision is incorrect or absent at all. It is characterized by a high threshold of agitation during head movements with significant acceleration. It is a kind of “defence” mechanism that protects a person against falling in a situation of loss of balance [15].

Other authors also believe that only performing a one leg test (itself) allows the functional assessment of the body control system. Haddas et al. searched for cone of the economy (CoE) during functional balance tests (Romberg's with eyes opened) in a group of adult degenerative scoliosis (ADS) patients [23]. ADS patients presented other control strategies compared to nonscoliotic controls, which involved more hip flexion and trunk flexion in a situation of loss of balance.

Because idiopathic scoliosis causes deformations of the body artery and, as many studies indicate, affects body balance [8, 16, 18, 19, 23–25], we decided to take a closer look at the mechanisms controlling posture.

The primary findings of our study are as follows:

1. In this study, statistical differences were found between the study group and control group in the mean postural control index and proprioceptive control index between the test and control groups. However, for the trials with eyes closed, the difference was much larger and could indicate deterioration of proprioceptive sensation. The results presented in the work indicate a significant reduction in proprioceptive sensation among patients diagnosed with idiopathic scoliosis. The proprioception index was significantly lower, comparing with people without curvature of the spine, which could indicate that proprioception is not the dominant mechanism in the body posture control process in this group of patients. Our results indicate that for patients with scoliosis, the body schema, which is based on a properly functioning proprioceptive system, is disturbed. Patients may experience disturbances in the sense of the mutual arrangement of body elements and the movement of its sections against each other. Thus, exercising without eye control may not be effective. We would like to draw the readers' attention to the fact that proprioception disorders may extend the time of rehabilitation and reduce its effectiveness. Exercises should be performed with the controlled body position not only by the person doing exercises but also by the physiotherapist and the patient's parents. Proprioception disorders increase the role of the parent in the process of conservative rehabilitation of scoliosis. It is also important to provide adequate visual feedback on body positioning during exercise, for example, using mirrors. Moreover, according to the authors [25, 26], proprioception plays a key role in maintaining the physiological function of the joints. Idiopathic scoliosis may derange the synchronization of static and dynamic mechanisms responsible for maintaining proper neuromuscular control, disrupting the function of the musculoskeletal system and joint function [27]. Therefore, it seems reasonable, that patients with scoliosis may be prone to joint injuries; that is why physiotherapeutic activities should also include exercises that strengthen joint stabilization and ensure their proper functioning
2. The authors of the study were unable to obtain statistically significant differences in the level of indexes of postural control and proprioceptive control in the subjects, depending on the value of the angle of scoliosis determined by the Cobb angle technique. The scoliosis magnitude does not determine the decrease in stability indexes. However, all patients presented a significant overall difference in results between the mean value of the stability index for open and closed eyes. The differences between the stability index levels in OE and CE trials inform about the influence of visual dependence on postural control. It was observed that patients with scoliosis showed a significantly higher dependence on the use of visual system in the body posture control process, which would point to an extremely important role of visual information in maintaining posture stability. Vision, whose major function is maintaining a relatively good balance with eyes open, may not be a sufficient component in the postural control. Impaired vision or any movement performed without the control of vision may result in a fall and injury. Moreover, regaining balance without visual control may be carried out inappropriately. Improving stance stability based on proprioceptive reflexes and with decreased visual dependence is crucial in preventing falls

Discrepant results were observed by Haumont et al. who observed that the amplitude of spine deformity in adolescent idiopathic scoliosis was a significant factor affecting the posture parameters [28]. Poorer postural control, which was observed especially in patients with a Cobb angle greater or equal to 15 degrees, reflects less effective central information processing. What is more, Ostrowska et al. points to the fact that regaining postural balance, after it has been disturbed, is considerably harder in children and adolescents with progressive scoliosis, and the volume and character of responses to regain postural control depend on the spine deformity [29]. The most significant alterations in maintaining a stable posture while regaining balance were noted in children with scoliosis exceeding 40 degrees, whereas in subjects with smaller scoliosis, the differences were very slight. No significant differences were observed in children with appropriate posture and mild scoliosis.

On the other hand, the results presented by Assaiante et al. show that the occurrence of spinal curvature does not affect the control of vertical orientation and body stabilization strategies in the group of patients with scoliosis [30]. In contrast, the authors suggest the existence of different afferent pathways for proprioceptive information, whose primary task is sensory integration of posture control. The authors emphasize that idiopathic scoliosis does not affect the static proprioceptive system (posture stabilization) but mainly affects the dynamic proprioceptive system (body's orientation). Vision improves posture control in both healthy adults and the ones with scoliosis, suggesting that vision plays a predominant role in adolescents' control.

Given the above article, we believe that drawing the right conclusions from many studies is only possible if the conditions under which the tests are conducted are harmonized. The fact whether the test is conducted under static or dynamic conditions, or position included standing on one or both limbs, can significantly affect the test results. The standardization of the study conditions is noticed by Dalleau et al., who, as in the present study, also proved that in the group of girls with scoliosis during quiet standing, there is a greater variation in the location of the foot pressure center compared to girls without scoliosis [31]. In the Beaulieu study, as in the Dalleau study, COP was used. The Beaulieu study showed that the group of girls with idiopathic scoliosis had inferior postural control, which affected the increased range of COP [32].

(3) No correlation was observed between the value of the ATR and the level of index of postural control with eyes open. For patients with scoliosis, appropriate postural control is obtained during single-leg standing with eyes open when the visual component used to maintain balance is greater. However, the authors of this study observed a significantly negative correlation between the level of index of proprioceptive control (single-leg standing with eyes closed) and the grade of trunk rotation examined by means of Bunnell scoliometer during the test of forward bending (ATR)

Testing torso rotation by means of a Bunnell scoliometer allows for objective assessment of a child's growing spine, and early detection of its rotation relative to the longitudinal axis of the body prevents further progression of scoliosis [33–35]. Samuelsson and Noren reached very interesting conclusions. They determined the angle of trunk rotation corresponding to specific values of lateral inclination of the vertebrae according to the Cobb method [36]. The criterion for the angle of trunk rotation of 7 degrees for thoracic scoliosis and 6 degrees for thoracolumbar and lumbar scoliosis, which has a curvature angle equal to or greater than 25 degrees of Cobb angle, was considered statistically significant. They noticed the need for further imaging of the spine of patients in whom screening, using a Bunnell scoliometer, showed that the trunk rotation angle was 6 degrees. What is more, in our opinion, it seems reasonable to assess body balance parameters on stabilometric platforms in patients with the angle of trunk rotation above 6 degrees. Its low result of the postural control parameters may additionally indicate the need to introduce proprioceptive control training in the algorithm of physiotherapy.

### 4.1. Limitations of the Study

In this study, we determined parameters of the postural control in relation to the alterations in the spine curvature disorders: the severity degrees of deformity and trunk rotation magnitude. The location of the curvature and the number of arches in the curvature could also contribute to the deterioration of the equilibrium parameters. Therefore, further studies should be extended to identify the influence of AIS type on the postural stability control. In addition, most patients with scoliosis were conservatively treated during the study. It should be checked in the long run whether the undertaken treatment and its type (kinesiotherapy, braces) improves proprioception.

Furthermore, the study group has a predominant number of girls; hence, the conclusions of the study cannot be transferred to children of both sexes, but rather to the female population. Nevertheless, scoliosis affects girls more often.

## 5. Conclusions

Significantly lower values of the stability index during single-leg standing with eyes closed indicated balance impairment which is mainly connected with inadequate functioning of the proprioceptive system. The character of these alterations is much influenced by the degree of spine rotation. The level of scoliosis according to the Bogdanov classification does not affect the level of SI OE and SI CE. Thus, the value of rotation of the trunk may be a factor considerably aggravating proprioceptive control. Therapeutic activities aiming at reducing the angle of the trunk rotation may result in the improvement of the person's balance.

## Figures and Tables

**Table 1:** Subject characteristics.

Variables No. of subjects	Study group			Control group			*p*
	Girls	Boys	All subjects	Girls	Boys	All subjects	
	69	11	80	21	19	40	
Age (years)	13.81 ± 1.76	15 ± 1.79	13.98 ± 1.8	14.48 ± 2.29	13 ± 1.07	14.11 ± 2.08	>0.05
Risser sign	2.31 ± 1.76	2.86 ± 1.19	2 ± 1.7	—	—	—	—
Height (cm)	161.83 ± 8.45	171.27 ± 10.57	163.13 ± 9.2	163.35 ± 13.85	162.75 ± 10.93	162.5 ± 12.6	>0.05
Body weight (kg)	48 ± 7.86	57.27 ± 9.56	49.27 ± 8.67	53.13 ± 12.76	55.25 ± 17.97	53.36 ± 13.65	>0.05
Body mass index (kg/m^2^)	18.28 ± 2.42	19.42 ± 1.87	18.43 ± 2.38	19.76 ± 2.38	20.40 ± 3.3	19.96 ± 3.3	>0.05

Values are mean ± SD.

**Table 2:** Group characteristics according to the value of the primary curve among 80 patients with idiopathic scoliosis according to Bogdanov.

Variables	Scoliosis value (°)		
	<20	20-40	41-60
No. of subjects (%)	21 (26)	47 (59)	12 (15)
Age ± SD (y)	13.42 ± 1.43	14.21 ± 1.69	14 ± 2.59
Cobb angle of the primary curve ± SD (°)	14 ± 2.59	28.89 ± 1.69	48.17 ± 2.59

Values are mean ± SD; SD: standard deviation; y: years.

**Table 3:** Characteristics of the participants from the study group in relation to the angle of trunk rotation (*n* = 79).

Variables	Study group		
	Group 1	Group 2	Group 3
Range of ATR value (°)	≤5	6-10	≥11
No. of subjects (%)	18 (23)	46 (58)	15 (19)
Age ± SD (y)	14.22 ± 1.73	14.07 ± 1.71	13.67 ± 1.92
ATR ± SD (°)	4.56 ± 1.73	8.94 ± 1.71	13.53 ± 1.92

ATR value: the angle of trunk rotation; values are mean ± SD; SD: standard deviation; y: years.

**Table 4:** Table showing multiple variables SI OE and SI CE in the study group with scoliosis divided with respect to the value of the angle of the trunk rotation. Three groups distinguished depending on the angle of trunk rotation: I, II, and III. Group I: ≤5° ATR, group II: 6-10° ATR, and group III: ≥11° ATR.

Variables	Group I	Group II	Group III	(pI, pII, pIII)
SI OE	86.45 ± 13.3	87.99 ± 8.4	84.77 ± 14.5	(NS, NS, NS)
SI CE	73.76 ± 27.8	72.82 ± 17.5	61.91 ± 30.4	(NS, <0.05, <0.05)

SI OE: the stability index in open eyes trials; SI CE: the stability index in closed eyes trials; pI: group I vs. group II; pII: group I vs. group III; pIII: group II vs. group III; NS: not significant.

## Data Availability

The date used to support the findings of this study may be released upon application to the Department of Rehabilitation Medicine, Medical University of Gdansk, who can be contacted at Aneta Dąbrowska; anetabytner@gumed.edu.pl.

## References

[1] Burke R. E. (2007). Sir Charles Sherrington’s. The integrative action of the nervous system: a centenary appreciation. *Brain*.

[2] Han J., Waddington G., Adams R., Anson J., Liu Y. (2016). Assessing proprioception: a critical review of methods. *Journal of Sport and Health Science*.

[3] Riva D. (2000). Archeopropriocezione. *Sport & Medicina*.

[4] Winter D. (1995). Human balance and posture control during standing and walking. *Gait & Posture*.

[5] Antoniadou N., Hatzitaki V., Stavridis S. Ι., Samoladas E. (2018). Verticality perception reveals a vestibular deficit in adolescents with idiopathic scoliosis. *Experimental Brain Research*.

[6] Hawasli A. H., Hullar T. E., Dorward I. G. (2015). Idiopathic scoliosis and the vestibular system. *European Spine Journal*.

[7] Pialasse J. P., Descarreaux M., Mercier P., Blouin J., Simoneau M. (2015). The vestibular-evoked postural response of adolescents with idiopathic scoliosis is altered. *PLoS One*.

[8] Konieczny M. R., Senyurt H., Krauspe R. (2013). Epidemiology of adolescent idiopathic scoliosis. *Journal of Children's Orthopaedics*.

[9] Bogdanov E. F. (1961). Idiopathic scoliosis. *Orvosi Hetilap*.

[10] Negrini S., Hresko T. M., O’Brien J. P., Price N., SRS Non-Operative Committee (2015). Recommendations for research studies on treatment of idiopathic scoliosis: consensus 2014 between SOSORT and SRS non-operative management committee. *Scoliosis*.

[11] Bunnell W. P. (1993). Outcome of spinal screening. *Spine*.

[12] Bunnell W. P. (1984). An objective criterion for scoliosis screening. *The Journal of Bone & Joint Surgery*.

[13] Riva D., Rocca F., Benedetti M. G. (2019). Effects of high-frequency proprioceptive training on single stance stability in older adults: implications for fall prevention. *BioMed Research International*.

[14] Riva D., Bianchi R., Rocca F., Mamo C. (2016). Proprioceptive training and injury prevention in a professional men's basketball team: a six-year prospective study. *Proprioception and Injury Preventation*.

[15] Riva D., Mamo C., Fanì M. (2013). Single stance stability and proprioceptive control in older adults living at home: gender and age differences. *Journal of Aging Research*.

[16] Dayer R., Haumont T., Belaieff W., Lascombes P. (2013). Idiopathic scoliosis: etiological concepts and hypotheses. *Journal of Children’s Orthopaedics*.

[17] Liu T., Chu W. C. W., Young G. (2008). MR analysis of regional brain volume in adolescent idiopathic scoliosis: neurological manifestation of a systemic disease. *Journal of Magnetic Resonance Imaging*.

[18] Gauchard G. C., Lascombes P., Kuhnast M., Perrin P. P. (2001). Influence of different types of progressive idiopathic scoliosis on static and dynamic postural control. *Spine*.

[19] Fortin C., Pialasse J. P., Knoth I. S., Lippé S., Duclos C., Simoneau M. (2019). Cortical dynamics of sensorimotor information processing associated with balance control in adolescents with and without idiopathic scoliosis. *Clinical Neurophysiology*.

[20] Wu K. W., Lu T. W., Lee W. C. (2020). Whole body balance control in Lenke 1 thoracic adolescent idiopathic scoliosis during level walking. *PLoS One*.

[21] Tobis J. S., Reinsch S., Swanson J. M., Byrd M., Scharf T. (1985). Visual perception dominance of fallers among community-dwelling older adults. *Journal of the American Geriatrics Society*.

[22] Riva D., Trevisson P., Minoletti R., Venturin N., Riccio M. (2001). Static and dynamic postural control in single stance. *Il Fisioterapista*.

[23] Haddas R., Satin A., Lieberman I. (2020). What is actually happening inside the "cone of economy": compensatory mechanisms during a dynamic balance test. *European Spine Journal*.

[24] Şahin F., Urak Ö., Akkaya N. (2019). Evaluation of balance in young adults with idiopathic scoliosis. *Turkish Journal of Physical Medicine and Rehabilitation*.

[25] Lanthier J., Simoneau M., Knoth I. S., Lippé S., Bluteau C., Fortin C. (2020). Increased EEG alpha peak frequency in adolescents with idiopathic scoliosis during balance control in normal upright standing. *Neuroscience Letters*.

[26] Guillou E., Dupui P., Golomer E. (2007). Dynamic balance sensory motor control and symmetrical or asymmetrical equilibrium training. *Clinical Neurophysiology*.

[27] Bartonek A., Lidbeck C., Hellgren K., Gutierrez-Farewik E. (2018). Head and trunk movements during turning gait in children with cerebral palsy. *Journal of Motor Behavior*.

[28] Haumont T., Gauchard G. C., Lascombes P., Perrin P. P. (2011). Postural instability in early-stage idiopathic scoliosis adolescent girls. *Spine*.

[29] Ostrowska B., Rożek-Piechura K., Skolimowski T. (2006). Odzyskiwanie dynamicznej równowagi po zewnętrznych zaburzeniach postawy u dzieci z idiopatyczną skoliozą. *Ortopedia Traumatologia Rehabilitacja*.

[30] Assaiante C., Mallau S., Jouve J.-L., Bollini G., Vaugoyeau M. (2012). Do Adolescent idiopathic scoliosis (AIS) neglect proprioceptive information in sensory integration of postural control?. *PLoS One*.

[31] Dalleau G., Allard M. S., Beaulieu M., Rivard C. H., Allard P. (2007). Free moment contribution to quiet standing in able-bodied and scoliotic girls. *European Spine Journal*.

[32] Beaulieu M. (2009). Postural imbalance in non-treated adolescent idiopathic scoliosis at different periods of progression. *European Spine Journal*.

[33] Cheung J. P. Y., Luk K. D.-K. (2017). Managing the pediatric spine: growth assessment. *Asian Spine Journal*.

[34] Zheng X., Tang Y. J., Nee A. Y. C. (2014). A novel evaluation index for adolescent idiopathic scoliosis progression measurement and diagnosis. *Technology and Health Care*.

[35] Amendt L. E., Ause-Ellias K. L., Eybers J. L., Wadsworth C. T., Nielsen D. H., Weinstein S. L. (1990). Validity and reliability testing of the scoliometer. *Physical Therapy*.

[36] Samuelsson L., Noren L. (2009). Trunk rotation in scoliosis the influence of curve type and direction in 150 children. *Acta Orthopaedica Scandinavica*.

